# “We do anything to get alcohol”: A mixed methods approach to explore the intersections between alcohol use and HIV risk and infection amongst adolescent girls and young women from high HIV-burdened communities in South Africa

**DOI:** 10.1371/journal.pone.0341299

**Published:** 2026-05-18

**Authors:** Kate Bergh, Zoe Duby, Jeroen De Man, Nabila Ebrahim, Caroline Kuo, Carl Lombard, Petal Petersen Williams, Kim Jonas

**Affiliations:** 1 Health Systems Research Unit, South African Medical Research Council, Cape Town, South Africa; 2 Department of Psychology, University of Cape Town, Cape Town, South Africa; 3 Division of Social and Behavioural Science, School of Public Health, University of Cape Town, Cape Town, South Africa; 4 Wimmy (Pty) Ltd, Cape Town, South Africa; 5 Department of Family Medicine and Population Health, University of Antwerp, Antwerp, Belgium; 6 Department of Health Studies, American University, Washington District of Columbia, United States of America; 7 Department of Psychiatry and Mental Health, University of Cape Town, Cape Town, South Africa; 8 Biostatistics Research Unit, South African Medical Research Council, Cape Town, South Africa; 9 Division of Epidemiology and Biostatistics, Stellenbosch University, Cape Town, South Africa; 10 Mental Health, Alcohol, Substance Use and Tobacco Research Unit, South African Medical Research Council, Cape Town, South Africa; 11 Department of Health Sciences, Mental Health and Addiction Research Group, University of York, York, United Kingdom; 12 Institute for Life Course Health Research, Department of Global Health, Stellenbosch University, Cape Town, South Africa; University of Nairobi, KENYA

## Abstract

Despite national declines in HIV prevalence, adolescent girls and young women (AGYW) in South Africa continue to face a disproportionate burden of HIV infection. The syndemic theory provides a framework for understanding how interconnected epidemics such as alcohol use disorders, violence and HIV cluster together and reinforce each other. Using this theory, we aimed to test and explore the relationship between alcohol use and HIV risk and infection amongst AGYW in high HIV-burdened communities in South Africa. Data for this study were drawn from a mixed methods impact evaluation of a combination HIV prevention programme implemented across eight provinces in South Africa. A household survey was conducted among AGYW aged 15─24 years in the 12 intervention sub-districts selected by the Global Fund and 12 matching comparison sub-districts. Dried blood spot specimens were collected to test for HIV; alcohol use and sexual behaviours were reported through a self-completed electronic questionnaire. Generalised linear regression models were used to assess the relationship between alcohol use and HIV risk and infection as well as sexual risk behaviours and hazardous drinking. A subsample of 68 AGYW participated in qualitative in-depth interviews. Interview transcripts were translated into English and analysed using thematic analysis. Of the 5025 survey participants, 10% were living with HIV, and 35% engaged in hazardous drinking. No significant association was found between alcohol use and HIV risk or infection. However, a positive association was found between hazardous drinking and sexual violence (OR=1.51; 95% CI: 1.20─1.90), condomless sex (OR=1.25; 95% CI: 1.07─1.47), and condom counselling (OR=1.32; 95% CI: 1.14─1.53). Qualitative interview participants described how AGYW sought alcohol from older men, knowing that sex would be expected in exchange. This put them in high-risk situations where sexual violence was a concern and negotiating condom use became more challenging. These findings highlight the need for interventions that educate AGYW on the harmful effects of binge drinking and provide safer, healthier alternatives for recreation, particularly in low-resourced communities.

## Introduction

South Africa has the largest HIV epidemic in the world [1]. Although HIV prevalence has declined from 14.0% in 2017 to 12.7% in 2022, adolescent girls and young women (AGYW) aged 15─24 years are the population at highest risk of HIV infection in the country [1]. HIV prevalence among AGYW was approximately double that of their male counterparts (6.9% versus 3.5%) in 2022 [1]. Thus, AGYW are a population with a critical need for HIV prevention and treatment interventions.

According to the World Health Organization (WHO), alcohol consumption per capita, defined as litres of pure alcohol consumed per persons aged 15 years and older in one year, was considerably higher in South Africa (8.8) compared to the global average (5.5) and the African regional average (4.5) in 2019 [2]. In South Africa, alcohol consumption per capita is higher among individuals aged 15 and older who have a higher socio-economic status (SES), while binge drinking, defined as current drinkers who consume five or more standard drinks on one occasion, is more prevalent among individuals with a lower SES [3]. Binge drinking in South Africa is a widespread and socially normalised behaviour, with 54% of male drinkers and 35% of female drinkers reporting such behaviour in 2021 [4]. The highest levels of binge drinking occur among people aged 18─35 years with research suggesting that binge drinking may even be underestimated among adolescents as they reach the same blood alcohol concentration as adults sooner given their smaller body size [3,5,6].

Data indicates that alcohol use is the leading cause of death and disability amongst adolescents and young people aged 15─24 years globally [7]. Alcohol consumption and binge drinking are on the rise among young women worldwide, despite the fact that women experience the harmful health effects of binge drinking (including cirrhosis, menstrual problems and various cancers) more frequently and severely than men [8,9]. Binge drinking often occurs in social contexts and is linked to a range of adverse consequences including high-risk sexual behaviours, driving under the influence of alcohol, accidents, and increased vulnerability to violence and sexual assault [10]. Research suggests that the 18─25 years age group is particularly vulnerable to alcohol related sexual risk behaviours, especially among young people living in low socio-economic communities [11].

In South Africa, alcohol use among women is closely linked to socio-economic pressures, emotional stressors and gender inequalities. Research indicates that many women drink as a coping mechanism to manage emotions and navigate social interactions amid challenges such as poverty, unemployment and gender-based violence (GBV) [12–14]. Alcohol use and inequitable gender norms are also predictors of experiences of intimate partner violence (IPV) and GBV for women and perpetration by men [14–16]. The link between GBV and condomless sex as well as alcohol use and sexual risk behaviours including condomless sex, multiple sexual partners and transactional sex is well established in sub-Saharan Africa and South Africa, contributing to HIV transmission risk [14,15,17–19].

Syndemics occur when multiple health issues cluster within a population by person, place or time, worsening health outcomes [20]. The “SAVA syndemic” (substance abuse, violence and HIV/AIDS) is a term used to describe the interconnected and mutually reinforcing nature of these health issues [21]. The “SAVA syndemic” is closely linked to gender inequalities and mental health issues which are also highly prevalent in populations with heightened HIV risk [4,14]. Most research on the “SAVA syndemic” has been conducted in the United States [22] and among men and women in South Africa [14,15,23], however, there is limited literature focusing on alcohol use and HIV risk among AGYW in South Africa [17,24]. Specifically, the pathways that explain the relationship between alcohol use and HIV risk among AGYW are not well understood in South Africa as well as the environments which foster the link between alcohol use and HIV risk, highlighting the need for mixed methods research to explore these mechanisms. This study applies syndemic theory to a mixed methods analysis of the co-occurrence of hazardous drinking and HIV amongst South African AGYW. We aimed to examine the relationship between hazardous drinking and HIV risk and infection as well as explore the overlapping and intersecting pathways that drive these syndemics among AGYW in high HIV-burdened communities in South Africa.

## Methods and materials

The data for this study were drawn from a mixed methods impact evaluation of the My Journey Programme, a combination HIV prevention programme implemented in 12 sub-districts in South Africa, considered to have high HIV prevalence and unintended pregnancy, since 2016. The programme aimed to reduce HIV incidence, teenage pregnancy, and GBV, and increase retention in school and access to economic opportunities. Core and layered services were provided in schools, Technical Vocational Education and Training (TVET) colleges, dedicated Safe Spaces and mobile clinics. Core services included enrolment and consent, an HIV risk and vulnerability assessment, and a service plan, while biomedical, behavioural and structural services were offered based on HIV risk as part of the layered services. The programme was implemented by South African civil society organisations, and the evaluation was conducted by the Health Systems Research Unit at the South African Medical Research Council. Both the programme and its evaluation were funded by the Global Fund to Fight AIDS, Tuberculosis and Malaria [25].

Only post-intervention data from the household survey and qualitative interviews were used in this study.

### Household survey

#### Sampling and data collection.

Between 24 January and 15 May 2024, we conducted a “post-intervention” survey in 24 sub-districts across eight provinces in South Africa, emulating a non-randomised cluster controlled trial. The study comprised four hierarchical levels of design and sampling. At the first level, 24 sub-districts (12 intervention and 12 matching comparison sub-districts) were assigned and selected, followed by the second level of targeted sites consisting of small area layers (SALs) with two sites selected per sub-district, generating a total of 48 sites. At the third level, dwellings (potential households) were sequentially selected and visited from a randomised list of dwellings in a SAL to determine if any AGYW lived there. At the final and fourth level, eligible participants were consented and interviewed across the targeted SALs of a site, with a sample size target of 100 participants, generating a planned total sample of 4800 AGYW.

**Level one:** The intervention sub-districts included all 12 intervention sub-districts where the My Journey Programme had been implemented. Matching comparison sub-districts were selected to be comparable in terms of demographics and HIV prevalence in 2015/16 [26–28] before the intervention began and could not be saturated with similar programmes that may have influenced the evaluation’s primary outcome, HIV prevalence.

**Level two:** Two sites were purposefully selected within each intervention and comparison sub-district by consulting the programme implementers to identify sites where the intervention was implemented, and consulting Geographic Information System based information to identify equivalent sites in the comparison sub-districts. A SAL is a geographical unit made up of one or more enumeration areas with a population of less than 500. All SALs within each site were assessed for suitability of sampling using available spatial data from the Stats-SA Geographic Information Frame (https://www.statssa.gov.za/). All non-residential (industrial, recreational, commercial etc.) areas were removed from the sample. The resultant SALs were assessed using the latest Stats-SA population figures (specifically population group, age, gender, employment) to identify SALs with suitable cluster sizes.

**Level three:** Dwellings (potential households) were considered suitable if there were eligible AGYW living there.

**Level four:** AGYW aged 15─24 years were eligible for the study if they resided in the selected household and consented to all aspects of the study. Participants were not eligible to participate if they had cognitive or mental challenges, were deaf or mute, or did not speak any of the study languages.

Upon consent, dried blood spot (DBS) specimens were collected from participants to test for HIV while socio-demographics, alcohol use, sexual behaviour and use of HIV prevention methods were reported through a self-completed electronic questionnaire with audio-assistance. The questionnaire was available in 10 of South Africa’s 11 official languages, predominant in the study areas.

#### Measures.

Three separate regression models were tested for this study. The **dependent variables** for these three models were 1) *current HIV risk, 2) HIV status* and *3) hazardous drinking*. *Current HIV risk* was a comprehensive predictive risk score for HIV developed using data from the baseline evaluation for the My Journey Programme, which included almost identical measures to the survey used for the HERStory3 Impact Evaluation [29]. The score was made up of a range of socio-demographic factors as well as sexual risk behaviours which are known to influence HIV risk. It was reported to have a positive predictive value of 68.2% and a negative predictive value of 85.8%. The score was expressed as a probability ranging from 0 to 1. Table 2 describes the variables we used for this score, with minor adaptations related to their categorisation. Relative SES included in the *current HIV risk* score was created using cluster analysis with the K-Modes algorithm [30]: three ordered subcategories were created from 13 SES related variables [31]. *HIV status* was confirmed via two separate enzyme-linked immunosorbent assays of DBS specimens collected. *Hazardous drinking* was determined using the shortened 3-item Alcohol Use Disorders Identification Test – Consumption (AUDIT-C) score [32,33], with a cutoff of 2 or more indicating hazardous drinking. We chose this cutoff because the literature recommends a cutoff of at least 2 for women [33], but since AGYW are less likely to be current drinkers than older women in South Africa [34], we thought that a cutoff at the lower limit would be appropriate and this cutoff has been used in another study among AGYW in South Africa [17]. The three questions of the AUDIT-C score included: 1) How often do you have a drink containing alcohol? 2) How many drinks containing alcohol do you have on a typical day when you are drinking? 3) How often do you drink 6 or more drinks on one occasion? The total score on the combination of these questions ranges from 0 to 12.

**Table 1:** Participant characteristics, alcohol use, HIV status, and sexual risk behaviours.

Variable	Option	15─19 years	20─24 years	Total
		Freq (%)	Freq (%)	Freq (%)
**Study arm**	Intervention	1514 (51.1%)	1124 (54.5%)	2638 (52.5%)
	Comparison	1447 (48.9%)	940 (45.5%)	2387 (47.5%)
**Relative socio-economic status**	Lower	810 (27.4%)	620 (30.0%)	1430 (28.5%)
	Middle	894 (30.2%)	530 (25.7%)	1424 (28.3%)
	Higher	1257 (42.5%)	914 (44.3%)	2171 (43.2%)
**Enrolled in education**	Yes	2304 (77.8%)	865 (41.9%)	3169 (63.1%)
	No	342 (11.6%)	843 (40.8%)	1185 (23.6%)
	Missing	315 (10.6%)	356 (17.2%)	671 (13.4%)
**Maternal orphanhood**	Yes	365 (12.3%)	445 (21.6%)	810 (16.1%)
	No	2562 (86.5%)	1602 (77.6%)	4164 (82.9%)
	Missing	34 (1.1%)	17 (0.8%)	51 (1.0%)
**Ever had sex**	Yes	1321 (44.6%)	1825 (88.4%)	3146 (62.6%)
	No	1465 (49.5%)	129 (6.2%)	1594 (31.7%)
	Missing	175 (5.9%)	110 (5.3%)	285 (5.7%)
**Hazardous drinking (AUDIT-C ≥ 2)**	Yes	819 (27.7%)	917 (44.4%)	1736 (34.5%)
	No	1762 (59.5%)	867 (42.0%)	2629 (52.3%)
	Missing	380 (12.8%)	280 (13.6%)	660 (13.1%)
**Hazardous drinking (AUDIT-C ≥ 3)**	Yes	627 (21.2%)	746 (36.1%)	1373 (27.3%)
	No	1954 (66.0%)	1038 (50.3%)	2992 (59.5%)
	Missing	380 (12.8%)	280 (13.6%)	660 (13.1%)
**DBS-confirmed HIV positive**	Yes	181 (6.1%)	320 (15.5%)	501 (10.0%)
	No	2720 (91.9%)	1711 (82.9%)	4431 (88.2%)
	Missing^1^	60 (2.0%)	33 (1.6%)	93 (1.9%)
**Sexual violence**	Yes	391 (13.2%)	287 (13.9%)	678 (13.5%)
	No	2088 (70.5%)	1473 (71.4%)	3561 (70.9%)
	Missing	482 (16.3%)	304 (14.7%)	786 (15.6%)
**Condomless sex**	Yes	695 (23.5%)	910 (44.1%)	1605 (31.9%)
	No^2^	2041 (68.9%)	959 (46.5%)	3000 (59.7%)
	Missing	225 (7.6%)	195 (9.4%)	420 (8.4%)
**Transactional sex**	Yes	267 (9.0%)	354 (17.2%)	621 (12.4%)
	No^2^	2440 (82.4%)	1416 (68.6%)	3856 (76.7%)
	Missing	254 (8.6%)	294 (14.2%)	548 (10.9%)
**Languishing**	Yes	378 (12.8%)	255 (12.4%)	633 (12.6%)
	No	2583 (87.2%)	1809 (87.6%)	4392 (87.4%)
**Condom counselling**	Yes	895 (30.2%)	996 (48.3%)	1891 (37.6%)
	No	1804 (60.9%)	928 (45.0%)	2732 (54.4%)
	Missing	262 (8.8%)	140 (6.8%)	402 (8.0%)

Note. The response options of “I don’t know” and “Prefer not to answer” were recoded as missing. ^1^Data for DBS was missing for 93 participants due to destroyed DBS specimens, challenges withdrawing blood from participants or insufficient sample on the DBS card. ^2^Only participants who reported ever having sex were asked about condomless and transactional sex. Participants who reported they had never engaged in sexual activity were included in the group answering “No”.

**Table 2:** Predictors for current HIV risk score.

Predictor	OR^2^ (95% CI)	p-value
Intercept^1^	0.04 (0.02─0.07)	<0.001
**Age**		
15-19 years	0	
20-24 years	2.35 (1.82─3.02)	<0.001
**Age at first sex**		
≤15 years	0	
>15 years	0.81 (0.59─1.11)	0.195
**Education**		
None or primary	0	
Secondary	0.84 (0.58─1.21)	0.349
Post-secondary	0.77 (0.48─1.24)	0.288
**Relative socio-economic status**		
Lower	0	
Middle	1.48 (1.13─1.93)	0.004
Higher	1.05 (0.81─1.35)	0.712
**Relationship status**		
Single	0	
Dating	1.36 (1.02─1.82)	0.037
Living together	1.18 (0.74─1.88)	0.480
**Last male sexual partner five or more years older**		
No	0	
Yes	1.75 (1.33─2.31)	<0.001
No partner	0.75 (0.54─1.04)	0.088
**Any symptoms of a sexually transmitted infection**		
No	0	
yes	0.99 (0.76─1.27)	0.908
**Current male partner HIV status**		
HIV negative	0	
HIV positive	9.88 (6.62─14.73)	<0.001
Don’t know	2.10 (1.56─2.82)	<0.001
No partner	2.54 (1.75─3.69)	<0.001
**Used condoms at last sex**		
No	0	
Yes	1.50 (1.20─1.87)	<0.001
**Ever engaged in transactional sex**		
No	0	
Yes	0.87 (0.65─1.17)	0.362
**One parent dead**		
No	0	
Yes	1.51 (1.23─1.85)	<0.001
**Ever pregnant**		
No	0	
Yes	1.14 (0.89─1.46)	0.290
**Ever sexually abused**		
No	0	
Yes	1.14 (0.86─1.51)	0.367

^1^Corresponds to the average odds when all predictor variables are zero. ^2^Quasibinomial odds ratio (OR) estimates and their 95% confidence intervals (95% CI) accounting for clustering at the level of the household, the site and the sub-district.

The *AUDIT-C score* was the **independent variable** for Model 1 and 2. The **independent variables** for Model 3 were *sexual violence, condomless sex, transactional sex, languishing* and *condom counselling. Sexual violence* was defined as having ever been sexually abused by a male partner or other men who were not the participant’s partner. *Condomless sex* was defined as having not used a condom throughout sex the last time you had sex with a boy or man. *Transactional sex* was defined as having oral, vaginal or anal sex in the past six months because you expected to get money or goods. *Languishing* is a measure of low levels of emotional, psychological and social wellbeing as defined by the Mental Health Continuum – Short Form (MHC-SF) [35,36]. *Condom counselling* was defined as having ever received instructions or counselling on how to use condoms.

To prevent confounding, the following **covariates** were added to all three models: *age, study arm, enrolled in education, maternal orphanhood, ever had sex,* and *relative SES* (defined above).

#### Data analysis.

Survey data from both intervention and comparison sites were pooled to test the hypothesised associations. Frequencies and percentages for dependent and independent variables included in the regression analyses were reported for the full survey sample. Three quasibinomial generalised linear models were used to test the association between the dependent and independent variables outlined above, accounting for nested clustering at the household, site and sub-district levels. The first model tested the relationship between *AUDIT-C scores* and *current HIV risk* among participants who were DBS-confirmed HIV negative. The second model tested the relationship between *AUDIT-C scores* and *HIV status* among participants with available DBS data*.* The third model tested the relationship between hazardous drinking (AUDIT-C score ≥ 2) and sexual violence, condomless sex, transactional sex, languishing, and condom counseling among all participants. All models accounted for potential confounding through inclusion of the covariates described under *Measures*. Response options “I don’t know” and “Prefer not to answer” were recoded as missing. To address this in the analysis, multiple imputation was performed under the missing-at-random (MAR) assumption. Imputations (m = 10) were generated through an expectation-maximisation with a bootstrapping (EMB) algorithm while maintaining grouping by the cluster variables. Pooling was done using Rubin’s rules, which account for both within-imputation variance and between-imputation variance. Analyses were carried out in R [37], using the “Survey” and “Amelia” packages [38,39].

### Qualitative interviews

#### Data collection and analysis.

Qualitative in-depth interviews were conducted with AGYW aged 15─24 years, from across the following intervention sub-districts: AbaQulusi and uMhlathuze (KwaZulu Natal), Mbombela and Govan Mbeki (Mpumalanga), Nelson Mandela Bay and Nyandeni (Eastern Cape), Dihlabeng and Setsoto (Free State), Tshwane 1 (Gauteng), Rustenburg (North West), and Klipfontein (Western Cape). The electronic survey was programmed with a prompt which appeared at the end of the consent form, and asked participants to provide their cell phone number if they would like to be invited to participate in one or more phone interviews. Participant recruitment for the qualitative interviews started on the 13^th^ of March 2024. Between 17 March and 3 May 2024, semi-structured qualitative interviews were conducted by female interviewers who were fluent in the study languages, with qualifications ranging from a Bachelor’s to a Masters’ degree and had received study specific training as well as training in research ethics. Interviewers were allocated according to their language skills and the dominant language spoken at each site (isiZulu, Sesotho, Setswana, Afrikaans, isiXhosa or English) and had no prior relationships with participants. Interviews were conducted telephonically using Teams software, were each approximately one hour in length, and were audio recorded with participants’ consent. Interviews followed topic guides that included questions on alcohol use such as asking participants to describe their views on alcohol use amongst young women in their community and in their peer group specifically, and own personal experiences with alcohol use, including the settings in which young women usually consume alcohol, the people young women drink alcohol with, how they access it, how alcohol affects their behaviour and emotions, and the impact alcohol has on their lives.

Interviews were audio-recorded and directly translated from their original language into English transcripts. Thematic analysis was conducted collaboratively by three analysts and followed an integrated and cyclical process using a set of pre-determined deductive code types based on the topics included in the interview guides, which were built upon through the inductive development and refinement of codes. The analysis process evolved iteratively through a deductive and inductive process reflecting the study’s key objectives and topics that emerged through reading the data. During the early stages of data collection, a set of preliminary themes and topic areas were defined based on the key research questions. The analysis structure aligned with the interview guide topics, while additional themes were identified iteratively through reading of the transcripts. In addition to descriptive themes, pattern themes, which achieve a greater level of abstraction, were used to start linking themes and topics together in order to explore relationships in the data.

### Ethics approval

Ethical approval for the study was granted by the Human Ethics Research Committee at the South African Medical Research Council (EC027-8/2023). All participants provided informed consent. For the electronic survey, written (digital) consent was obtained and signed consent forms are stored on a password protected computer. For the telephonic interviews, verbal consent was audio-recorded and is stored on a password protected computer. For participants under 18 years of age, parental/guardian consent was obtained. Participants received a R200 (US$ 11) reimbursement for their participation.

## Results

### Quantitative findings

Across study arms, 37,714 dwellings were visited, 22,263 households were screened, 5150 AGYW were invited and 5025 participated (97.5% response rate) (i.e., the sample used for Model 3). Of the 5025 survey participants, 4932 (98.2%) had available DBS data (sample used for Model 2) and 4431 (88.2%) were DBS-confirmed HIV negative (sample used for Model 1).

Table 1 describes participant characteristics, alcohol use, HIV status and sexual behaviour among all study participants (n = 5025), stratified by age group (15─19 years: 58.9%; 20─24 years: 41.1%). There were 501 (10.0%) participants who were DBS-confirmed HIV positive and 1736 (34.5%) who engaged in hazardous drinking (AUDIT-C ≥ 2).

Table 2 describes the variables included in the current HIV risk score, which is the outcome variable for the first regression model described in Table 3. Several variables in this score were not significantly associated with the score including age at first sex, education, any STI symptoms, ever engaged in transactional sex, ever pregnant and ever sexually abused.

**Table 3:** Association between AUDIT-C score and 1) current HIV risk and 2) HIV status.

Predictors	Outcome: Current HIV risk		Outcome: HIV status	
	OR^2^ (95% CI)	p-value	OR^2^ (95% CI)	p-value
Intercept^1^	1.46 (0.99─2.15)	0.059	0.00 (0.00─0.01)	<0.001
AUDIT-C Score	0.99 (0.98─1.02)	0.654	0.99 (0.95─1.03)	0.762
Age (in years)	0.99 (0.97─1.01)	0.464	1.21 (1.16─1.27)	<0.001
Study arm (ref: comparison)	1.02 (0.93─1.10)	0.726	1.05 (0.86─1.28)	0.624
Middle SES (ref: lower SES)	0.95 (0.85─1.05)	0.340	1.32 (1.03─1.70)	0.029
Higher SES (ref: lower SES)	0.94 (0.85─1.04)	0.258	0.92 (0.73─1.17)	0.490
Enrolled in education (ref: no)	0.97 (0.87─1.09)	0.634	0.98 (0.79─1.23)	0.872
Maternal orphan (ref: no)	1.10 (0.98─1.24)	0.102	1.70 (1.35─2.14)	<0.001
Ever had sex (ref: no)	1.19 (1.06─1.34)	0.003	1.23 (0.92─1.65)	0.163

^1^Corresponds to the average odds when all predictor variables are zero. ^2^Quasibinomial odds ratio (OR) estimates and their 95% confidence intervals (95% CI) accounting for clustering at the level of the household, the site and the sub-district.

As shown in Table 3, there was no evidence for a statistically significant association between participants’ AUDIT-C score and current HIV risk (OR=0.99; 95% CI: 0.98─1.02). Reporting that you had ever had sex was the only covariate associated with a higher HIV risk score (OR=1.19; 95% CI: 1.06─1.34).

There was also no evidence for a statistically significant association between participants’ AUDIT-C score and HIV status (OR=0.99; 95% CI: 0.95─1.03). The covariates of age (OR=1.21; 95% CI: 1.16─1.27), maternal orphanhood (OR=1.70; 95% CI: 1.35─2.14) and being in the middle SES group (OR=1.32; 95% CI: 1.03─1.70) were positively associated with being HIV positive.

When assessing the association between one specific sexual risk behaviour and hazardous drinking, controlling for socio-demographic factors only (age, study arm, SES, enrolled in education and maternal orphanhood), we found that sexual violence (OR=1.77; 95% CI: 1.48─2.12), condomless sex (OR=0.83; 95% CI: 0.71─0.96), transactional sex (OR=1.25; 95% CI: 1.03─1.52) and condom counselling (OR=1.49; 95% CI: 1.30─1.71) were all positively associated with hazardous drinking (AUDIT-C score ≥ 2). Languishing was not associated with hazardous drinking (OR=1.12; 95% CI: 0.89─1.40).

Table 4 presents us with the findings from the same association between sexual risk behaviours and hazardous drinking, but now with all sexual risk behaviours in one model, allowing adjustment for their interrelationships. We found that sexual violence (OR=1.51; 95% CI: 1.20─1.90), condomless sex (OR=1.25; 95% CI: 1.07─1.47) and condom counselling (OR=1.32; 95% CI: 1.14─1.53) were all positively associated with hazardous drinking (AUDIT-C score ≥ 2). The covariates of age (OR=1.04; 95% CI: 1.01─1.07), ever having sex (OR=3.62; 95% CI: 2.99─4.38) and being in the higher SES group (OR=1.67; 95% CI: 1.42─1.96) were positively associated with hazardous drinking, while being enrolled in education (OR=0.79; 95% CI: 0.67─0.94) was negatively associated with hazardous drinking.

**Table 4:** Association between sexual risk behaviours and hazardous drinking (AUDIT-C score ≥ 2).

Predictor	OR^2^ (95% CI)	p-value
Intercept^1^	0.08 (0.04─0.14)	<0.001
Sexual violence (ref: no)	1.51 (1.20─1.90)	<0.001
Condomless sex (ref: no)	1.25 (1.07─1.47)	0.007
Transactional sex (ref: no)	0.89 (0.72─1.09)	0.258
Languishing (ref: no)	1.04 (0.85─1.27)	0.712
Condom counselling (ref: no)	1.32 (1.14─1.53)	<0.001
Age (in years)	1.04 (1.01─1.07)	0.014
Study arm (ref: comparison)	1.06 (0.93─1.22)	0.364
Middle SES (ref: lower SES)	0.88 (0.73─1.06)	0.167
Higher SES (ref: lower SES)	1.67 (1.42─1.96)	<0.001
Enrolled in education (ref: no)	0.79 (0.67─0.94)	0.010
Maternal orphan (ref: no)	1.10 (0.91─1.33)	0.311
Ever had sex (ref: no)	3.62 (2.99─4.38)	<0.001

^1^Corresponds to the average odds when all predictor variables are zero. ^2^Quasibinomial odds ratio (OR) estimates and their 95% confidence intervals (95% CI) accounting for clustering at the level of the household, the site and the sub-district.

### Qualitative findings

The final qualitative sample comprised 68 AGYW, with 13 in the 15─17 years age group, and 55 in the 18─24 years age group. A total of 7 participants were from Kwa-Zulu Natal province, 14 from Mpumalanga, 3 from the Eastern Cape, 11 from the Free State, 8 from Gauteng, 10 from the North West and 15 from the Western Cape province. The findings below present data on analytic themes related to alcohol use, and other risk behaviours. Direct quotations from the English transcripts are presented in quotation marks, followed by participants’ study site and age group in brackets.

#### Prevalence of hazardous drinking.

Most interview participants perceived alcohol use among AGYW to be high and problematic and that AGYW drink excessively without thinking of the consequences: “When they get the chance to go out then they will drink like there is no tomorrow” (Mpumalanga – AGYW 18─24 years).

Respondents noted a rise in alcohol use among AGYW: “Nowadays girls have changed… they drink a lot, before it was known to be boys who misbehave this much towards alcohol and drugs, but now it’s the girls” (KZN – AGYW 18─24 years). Others mentioned that even very young teenagers are drinking: “even the thirteen year olds here in my community are drinking alcohol… it’s quick to get and children are really abusing it” (Western Cape – AGYW 18─24 years).

#### Consequences of AGYW alcohol use.

Respondents identified several negative consequences of AGYW alcohol use, including vulnerability to violence and an increased risk of having unsafe sex.

##### Violence:

Respondents suggested that when AGYW accept or request alcohol from men, they may find themselves in dangerous situations and pressured into engaging in risk behaviours: “We go out to clubs with no money with the hopes that someone will buy drinks for us… In return, they want to sleep with us and that increases the chances of getting HIV… **When we go to the tavern, we say yes to everything** so that the guys buy alcohol for us, when the tavern closes, the guy wants to leave with you and if you refuse you can get killed” (Mpumalanga – AGYW 18─24 years). Some respondents suggested that the desire for alcohol often outweighed concerns for personal safety: “we leave with people we do not know… they want to sleep with you… **you risk your life for alcohol**” (North West – AGYW 18─24 years).

Respondents described drinking in isolated locations, such as abandoned buildings: “we go a distance away from the houses. In my village there are houses encircled by trees, there are places that some guys would hang out… we normally would go there” (Eastern Cape – AGYW 15─17 years). They also highlighted the risks of socialising with dangerous and violent people: “they drink alcohol they fight after that… alcohol is very dangerous it can make you do different kind of stuff… they sit on the road, they sit by some yards… they usually drink with men… **young girls with big boyfriends**” (Western Cape – AGYW 18─24 years).

The theme of **alcohol use and sexual violence** also emerged in the context of AGYW seeking sex themselves and elevating their risk for experiencing violence or impairing behaviours that can prevent perpetration of violence such as communicating clear sexual consent: “Sometimes… girls end up doing things they aren’t supposed to do… they can’t control their hormones and throw themselves at boys… Some will end up being victims of rape” (KZN – AGYW 18─24 years). It was suggested that younger girls in particular struggle to control their behaviour after drinking, making them more vulnerable to sexual violence: “(drinking alcohol) is not good especially for girls who are underage and are still attending school, because if they get drunk, men may take advantage of her and rape her, because she’s drunk and has no control… the alcohol is too strong for them” (Free State – AGYW 15─17 years).

##### Unsafe sex:

Respondents explained that in some cases, AGYW get pregnant after having sex whilst drunk: “a lot of incidents happen because of alcohol… like getting pregnant and not knowing who’s the father or getting raped or something” (Western Cape – AGYW 15─17 years). Getting drunk and losing control leads to decreased sexual agency for negotiating condom use, which in addition to increasing HIV risk, also increases the risk of pregnancy: “Girls drink a lot… to a point where someone is unable to control themselves… boys can take advantage of them…then they get pregnant” (Free State – AGYW 18─24 years). Loss of control combined with alcohol related amnesia was described: “Girls in our community drink alcohol a lot, they do not use it minimal like other people. They drink a lot to an extent that they cannot tell what really transpired the day before… it is very bad” (Free State – AGYW 18─24 years).

##### Transactional sex:

The theme of entering into transactional interactions with men, often referred to as “sugar daddies”, in order to obtain alcohol was salient in the data: “We have many providers (of alcohol)… older than we are” (Free State – AGYW 18─24 years). Most often these transactional engagements are with older men: “We drink with (giggle) our boyfriends… you will find that most of our boyfriends there are soldiers and teachers, we mingle with such people… our boyfriends buy for us” (Free State – AGYW 18─24 years).

Importantly, AGYW were described as agentic in these transactional engagements to procure alcohol: “the girls that like to hang out in places that sell alcohol, they follow boys around because they want alcohol… boys buy alcohol for them... in return for something” (KZN – AGYW 18─24 years). In some cases, AGYW actively seek out men to purchase drinks for them: “(I usually drink at a) tavern near my home and a shebeen nearby… I meet men who will buy for me… I do ask them” (Mpumalanga – AGYW 18─24 years). Having their own money to purchase alcohol was described as unnecessary as AGYW can rely on men to buy alcohol for them: “Girls go to taverns even if they do not have money, but they will come back drunk because there are men and sugar daddies that will provide them with alcohol” (Mpumalanga – AGYW 18─24 years).

The transactional nature of these interactions appeared to be widely accepted and expected: “Girls drink free alcohol from men… They drink with men who buy them alcohol… because they want to have sex with them” (Free State – AGYW 15─17 years). However in some cases, despite men’s expectations of having sex in return for purchasing alcohol, sometimes AGYW choose not to follow through and comply with this expectation: “girls go to taverns with no one, expecting someone else to buy for them… Men that also drink in the taverns (buy for them)… in return the men want sex…But, the girls drink with them and run away after” (Mpumalanga – AGYW 18─24 years).

## Discussion

This study sought to test and explore the relationship between the overlapping and intersecting syndemics of HIV and alcohol use disorders among AGYW from high HIV-burdened communities in South Africa. Findings showed that HIV prevalence among participants was as high as 10% with more than one third of participants engaging in hazardous drinking. Although quantitative analyses did not find a direct association between hazardous drinking and HIV risk or infection, there was an association between hazardous drinking and sexual risk behaviours including sexual violence and condomless sex. In qualitative interviews, AGYW described a range of consequences of alcohol use among AGYW including increased risk behaviours such as sex whilst drunk, increased risk of having condomless sex, engaging in transactional sexual relationships with older men to obtain alcohol, increased vulnerability to sexual violence, and increased exposure to dangerous situations, people, and places.

Our findings suggest that the prevalence of hazardous drinking among our sample of AGYW was high and problematic (35%; AUDIT-C score ≥ 2). Evidence for the prevalence of hazardous drinking among AGYW in South Africa is limited, but a similar prevalence of 33% (AUDIT-C score ≥ 2) was reported among sexually active AGYW (n = 3009) in the baseline survey for this impact evaluation [17]. Other smaller studies among HIV negative AGYW enrolled in HIV prevention programmes in sub-Saharan African countries including South Africa have reported prevalences of 37% (n = 427) [40] and 39% (n = 247) [41], respectively, using an even stricter cutoff of 3 or more for the AUDIT-C score. Two studies in Zimbabwe and Uganda reported on alcohol use using the 10-item AUDIT score out of 40. In Zimbabwe, hazardous drinking (AUDIT score ≥ 8) was reported at 2% among AGYW aged 18─24 years [42], while in Uganda, high risk drinking (AUDIT score ≥ 16) was reported at 16% among AGYW aged 14─19 years [43], suggesting that the prevalence in South Africa is high for the region. Interview participants also expressed concerns that alcohol use was increasing among AGYW including younger adolescents. There is some evidence in the literature to suggest that alcohol use among adolescents in sub-Saharan Africa is increasing [44], and among AGYW in particular, as regular and heavy drinking becomes socially accepted and normalised among this population [45,46].

Despite the increased social acceptability of alcohol use among AGYW in South Africa and their vulnerability to HIV, we found no statistically significant association between hazardous drinking and HIV risk or infection. Although an association between alcohol use and HIV risk is inferred by many studies due to the link between alcohol use and sexual risk behaviours [14,15,17], there is very little evidence from sub-Saharan Africa to demonstrate an association between alcohol use and a comprehensive measure of HIV risk or biologically-confirmed HIV status. We found one nationally representative study among South African AGYW which showed that low risk alcohol use was associated with a decreased likelihood of being HIV positive [47] and another nationally representative study in Zambia and Zimbabwe which showed an association between males and females aged 15─49 years who drank alcohol and being HIV positive [48]. The lack of association observed in this study could also be due to the cross-sectional study design as the causal pathway from alcohol use to HIV infection may take time to unfold. However, our study did show an association between alcohol use and HIV status before adding covariates to the model, suggesting that the covariates of age, enrolled in education, maternal orphanhood and SES interfere with the relationship between hazardous drinking and HIV status. This is supported by a study of the general adult population in South Africa which found that the age-standardised HIV-related mortality rate attributable to alcohol use was significantly higher in low versus high SES groups [49]. In addition, an agent-based model of binge drinking, gender inequalities and their contribution to HIV incidence among South African adults reported that 6.8% of new HIV infections where attributable to binge drinking, mediated by casual sex, and 17.5% were attributable to gender inequalities, mediated by male partner concurrency [4]. This is further supported by findings that AGYW in age-disparate and transactional relationships, in which the gendered power dynamics are inherently unequal, are more likely to be living with HIV [50]. Thus, the pathways which explain the association between alcohol use and HIV risk and infection are complex, overlapping and intersecting with other socio-demographic and structural factors.

We found a positive association between sexual violence, from partners and non-partners, and hazardous drinking in the quantitative analysis, which was further supported and explained in the qualitative interviews. The qualitative results describe a unidirectional relationship between alcohol use and physical and sexual violence wherein AGYW experienced dangerous situations by asking older men for alcohol in exchange for sex. Both engaging in these transactional sexual acts as well as refusing to have sex in these situations put AGYW at risk of sexual violence. Younger AGYW were particularly vulnerable to being taken advantage of because they had a lower tolerance for alcohol. This supports findings from studies which found that alcohol use predicted physical and sexual IPV experiences by women and perpetration by men aged 18 years and older in South Africa [15,16,24]. Notably, one nationally representative study, which used data from the South African Demographic and Health Survey in 2016, found that harmful drinking and HIV clustered together with physical IPV, but not sexual IPV, although these results may have been influenced by the lower-than-expected levels of IPV reported in the survey [23]. Other studies have reported that experiences of physical and sexual abuse among women aged 18 and older predicted alcohol use, which these studies speculate could be because women who drink are likely to be in relationships with partners who drink or because women who have experienced IPV use alcohol as a coping mechanism [14,51]. Qualitative studies among AGYW as well as older female survivors of IPV (≥18 years) have also described alcohol use as a negative coping mechanism which helped them to manage emotions and escape their reality [13,45,46], but motivations for drinking among younger women may be more influenced by peers and a desire to have fun, suggesting that the bidirectionality of this relationship is more salient among older women.

In qualitative interviews, participants described the locations in which drinking occurs, which were often dangerous places which enhanced their vulnerability to violence and sexual assault. It is important to recognise that it is not just risk behaviours that can exacerbate risk, but physical spaces or risk environments. Globally, young women’s engagement with what is referred to as the “night-time economy” of drinking establishments, makes them vulnerable to sexual abuse and violence [52]. More specifically, links between increased sexual risk behaviours, alcohol consumption, exposure to IPV, heightened HIV risk, and frequenting establishments such as taverns and shebeens, amongst AGYW in sub-Saharan Africa has been documented, suggesting an overlapping syndemic relationship between them [14,24,53].

Condomless sex and hazardous drinking were also associated in our quantitative analyses, which the qualitative results explained were due to impaired sexual agency to negotiate condom use whilst drunk or being forced to have sex without a condom whilst drunk. In addition, participants described how they could not remember whether they had had condomless sex whilst drunk or not, which we speculate would make them less likely to take precautions such as using emergency contraception to prevent pregnancy and post-exposure prophylaxis to prevent HIV acquisition. The link between hazardous drinking and condomless sex is well established in the literature among AGYW in South Africa, which studies explain is linked to an impaired ability to negotiate condom use whilst drunk [5,14,17].

Based on our final model which included socio-demographic and other sexual risk behaviours as covariates, we found no association between transactional sex and hazardous drinking. However, a model without these other sexual risk behaviours showed a strong association between transactional sex and hazardous drinking. This suggests that these sexual risk behaviours influence each other’s relationship with hazardous drinking. For example, condomless sex might have acted as a mediator along the causal pathway and mistakenly removed the true association between transactional sex and hazardous drinking (overadjustment), or sexual violence, which can be caused by hazardous drinking and transactional sex, could have masked the relationship between both (collider bias). Alternatively, the lack of association between transactional sex and hazardous drinking in our final model could be because of the way the transactional sex variable was asked in the survey questionnaire. AGYW were asked if they had had oral, vaginal or anal sex in the past six months because they expected to get money or goods, but alcohol-related transactions may be episodic and more infrequent than six months among AGYW, and alcohol was not specifically listed as one of the goods.

The relationship between transactional sex and hazardous drinking underpinned our qualitative findings, specifically the provision of alcohol by older men to AGYW in exchange for sex. The transactional nature of these interactions appeared to be widely accepted and expected by participants in our study, concurring with previous evidence [17,53]. Transactional sex in exchange for alcohol is a well-documented phenomenon among AGYW in South Africa [17,42,54,55], but our finding that AGYW can be agentic in these transactional sexual relationships has only been explored by two other qualitative studies [17,55]. However, AGYW’s agency to successfully negotiate and engage in safe and consensual sex is still hindered by the gendered power differentials inherent in transactional relationships with older men, which are further enhanced when AGYW have been consuming alcohol [20]. Together with findings that AGYW in transactional and age-disparate relationships are more likely to be living with HIV, our findings suggest that alcohol may mediate the relationship between gendered power asymmetries and HIV risk [50]. While there is some evidence to support this pathway among the adult South African population [4,14], it has not been explicitly studied among AGYW in South Africa and could be an interesting focus for future studies.

Having low levels of wellbeing (languishing) was not associated with alcohol use in our quantitative analyses and mental health was not the focus of the qualitative data for this study. This is supported by findings among adolescent girls aged 16─19 years in South Africa which found no relationship between mental health, including measures of depression and anxiety, and alcohol use [56]. However, depression and anxiety are higher in subgroups of young women aged 18─30 years with higher levels of alcohol use [54], with one study showing how depression predicts alcohol use among older women (≥ 18 years) [16]. In addition, another qualitative study drawing from the same data set described the dual role of alcohol as both a coping mechanism and a driver of negative mental health outcomes, but still highlighted entertainment as the primary driver for alcohol use among this age group [46]. Other potential reasons for the lack of association between mental health and hazardous drinking could be that: 1) we used a measure of wellbeing and not depression or anxiety, 2) the wellbeing scale we used has not been validated among AGYW in South Africa and 3) social desirability bias.

While other studies have shown that education is a protective factor in the relationship between alcohol use and sexual risk behaviour among young women in South Africa [5,16], our study found a positive association between receiving instructions or counselling on how to use condoms and hazardous drinking in our quantitative analyses. We speculate that this is because the My Journey Programme provided condom counselling to AGYW who they deemed to be at a higher risk of HIV acquisition following the programme’s risk assessment which was conducted among all programme beneficiaries as part of the programme’s core services. Thus, we do not think the results suggest that condom counselling facilitates hazardous drinking, but rather that participants who were already hazardous drinkers, were offered condom counselling by the programme.

### Limitations

Limitations of this study are firstly that some of the variables in the current HIV risk score were not significantly associated with the score. Although the positive and negative predictive values for the score were relatively high when it was applied to the baseline survey data for the My Journey Programme, HIV prevalence in the HERStory3 survey data used for this study was lower (10.0% vs. 12.4%), reducing the number of HIV-positive cases and decreasing statistical power [29]. However, DBS-confirmed HIV status was also used as an outcome in this study to examine the relationship between HIV and alcohol use. Secondly, we were unable to test the direction of the relationships between alcohol use, HIV risk and infection, and sexual risk behaviour because the data is from a cross-sectional study. Nevertheless, our regression models support existing evidence of the association between hazardous drinking and sexual risk behaviour, where the bidirectionality of these relationships has previously been explored. Thirdly, we found no association between hazardous drinking and mental health, suggesting that AGYW may have different motivations for drinking compared to older women, thus the direction of the relationship remains unclear for this population. Fourthly, while both the electronic survey and qualitative interviews may be subject to social desirability bias, steps were taken to minimise this, including a comprehensive informed consent process. Participants were informed that there were no immediate benefits to them for participation in the survey or interview and that their information would be kept anonymous and confidential. Fifthly, the AUDIT-C score has not been validated among AGYW in low-resourced settings and there is inconsistency in the literature regarding whether a cutoff of 2 or more, or 3 or more, should be used to identify hazardous drinking among women [33,57]. We tested Model 3 using both cutoff points and found no statistically significant differences in the results. Finally, sub-districts and sites within the intervention and comparison arms were purposefully selected to optimise the programme effect in this non-randomised comparison. Systematic random sampling was used to select households within the selected sites, but at higher levels, sites and sub-districts were not selected based on a random sampling technique. As such, we cannot claim our sub-districts nor study participants to be representative for the general South African AGYW population. This must be considered when interpreting the descriptive statistics and prevalence estimates. However, we believe that after controlling for key covariates, the identified associations correspond to a general trend relevant to the target population of AGYW in high HIV-burdened communities.

## Conclusions

Our findings support the concept of the “SAVA syndemic”, highlighting the co-occurrence and overlap of alcohol use disorders, sexual violence, and other high-risk sexual behaviours that increase AGYW’s vulnerability to HIV acquisition. This underscores the need for combination HIV prevention programmes which address the complex social, structural and behavioural factors that increase AGYW’s risk of these syndemics. However, findings also support specific targeting of HIV prevention and care interventions for AGYW with problematic alcohol use.

Transactional sex emerged as a major theme, reflecting both the agency that AGYW exercised in entering such relationships and the heightened exposure to sexual violence and condomless sex that often followed. Interventions addressing hazardous drinking should encourage young people to identify harmful drinking patterns and assess the costs and benefits of alcohol consumption, promoting healthier decision making [54]. One South African study among AGYW emphasised the importance of interventions that facilitate conversations with AGYW about how binge drinking impairs their agency to negotiate condom use in relationships [5]. Equipping AGYW with the skills to negotiate their own safety and manage potential risks in environments where drinking occurs may be beneficial [5].

Addressing gender inequalities is crucial for preventing HIV acquisition in the context of heavy drinking. One study highlighted the need for interventions to improve financial empowerment among AGYW to reduce their engagement in transactional sexual relationships [5]. Another study among older males and females in South Africa identified a resilience pathway, showing that education can reduce IPV [58]. Boys and men should also be included in these educational programmes, as addressing inequitable gender norms and hegemonic masculinities is key to promoting consensual and safe sex, particularly in the context of heavy alcohol consumption [4].

Finally, AGYW were motivated to drink by a desire to have fun, and we found no link between alcohol abuse and mental health, which has been found among older women in South Africa [13,14]. Hence, it is important to consider the environments where young people congregate for fun and how they may enhance risk when designing interventions. Findings from this study, along with existing literature, highlight that syndemics often occur in specific high-risk settings, such as drinking establishments in South Africa [14]. To mitigate this risk, sexual and reproductive health (SRH) messaging and condoms should be provided at drinking establishments to promote use [19,49]. However, AGYW also need access to safer and healthier sources of recreation, which can be offered through community-based youth venues such as Safe Spaces, as provided by the My Journey Programme. Safe Spaces have been shown to be acceptable to AGYW who are motivated to utilise Safe Spaces by educational, employment related, and recreational activities, and who then often engage in SRH interventions once there [31]. School-based interventions may be a cost-effective approach for school-going AGYW and Safe Spaces providing SRH services within schools have shown to be highly acceptable to AGYW [4,59].
